# Intussusception Caused by Peutz-Jeghers Syndrome

**DOI:** 10.7759/cureus.23792

**Published:** 2022-04-03

**Authors:** Harsha S Sreemantula, Crystal A Joseph, Faraz Jamal, Shubham Agrawal, Rajesh Thirumaran

**Affiliations:** 1 Internal Medicine, Mercy Catholic Medical Center, Darby, USA; 2 Hematology/Oncology, Drexel University College of Medicine, Darby, USA; 3 Physical Medicine and Rehabilitation, Mercy Catholic Medical Center, Darby, USA; 4 Hematology/Oncology, Mercy Catholic Medical Center, Darby, USA

**Keywords:** anemia, endoscopic screening, small bowel obstruction, intestinal polyps, small bowel resection, peutz jeghers, recurrent intussusception

## Abstract

A 20-year-old female patient with a family history significant for Peutz-Jeghers syndrome presented to the hospital multiple times with complaints of abdominal pain. On the initial visit to the hospital, the patient underwent small bowel resection for small bowel obstruction secondary to intussusception, following which she visited the hospital again one year later for similar complaints and underwent reduction of multiple points of intussusception of the small bowel without any resection of the same. Eventually, the patient underwent resection of the small bowel for the second time, along with tumor resections. The importance of follow-up in patients with Peutz-Jeghers is particularly essential, in part, because it is vital to monitor the tumors, their size, and number to prevent surgical intestinal complications, anemia, and also to eventually monitor for carcinomatous changes.

## Introduction

Peutz-Jeghers syndrome (PJS) is a rare genetic disorder characterized by mucocutaneous pigmentation in the perioral and buccal mucosa, and hamartomatous polyps in the gastrointestinal tract commonly found in the small bowel [1]. PJS has an autosomal dominant inheritance, and genetic mutations of the serine-threonine kinase 11 coding gene (STK11) on chromosome 19p13 have been linked to PJS [2,3]. However, not all patients with PJS have this mutation. Prior studies have shown that patients with PJS are at an increased risk for gastrointestinal and extra-gastrointestinal malignancies, such as genital tract and breast cancers [3]. The incidence of cancer in PJS patients is approximately 15 times higher than that of the general population [4] . 

 It is important to note that gastrointestinal polyps, commonly seen in PJS, can cause complications such as acute GI bleeding, bowel obstructions, rectal prolapse, and intussusception [5]. Recurrent bowel obstruction and intussusception can lead to multiple small bowel or colonic resections, posing a risk for short-gut syndrome in these patients. By age 10, there is a cumulative intussusception risk of 15%, and by age 20, this risk increases to 50% [6]. Since PJS can cause various complications, screening is vital to prevent surgical interventions. Current surveillance recommendations include a colonoscopy, upper GI endoscopy, and extra-intestinal tumor screening [7].

## Case presentation

**Figure 1:**
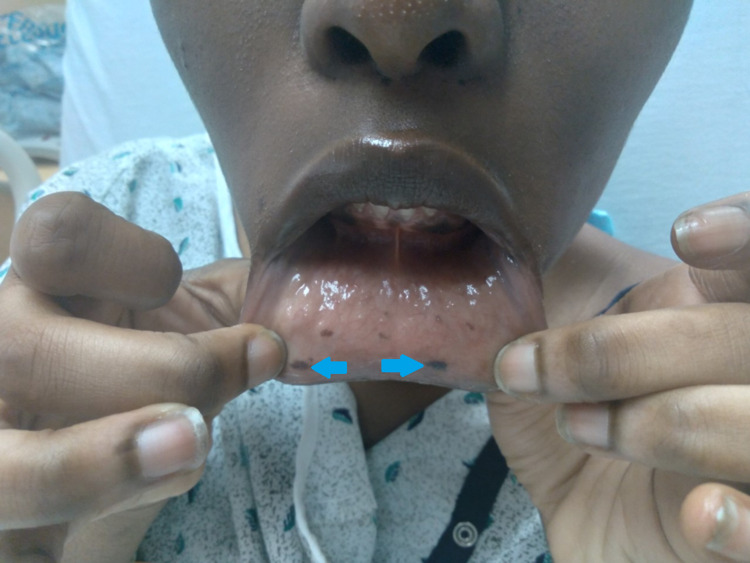
Mucocutaneous pigmented lesions on the lower oral mucosa and perioral skin

**Figure 2:**
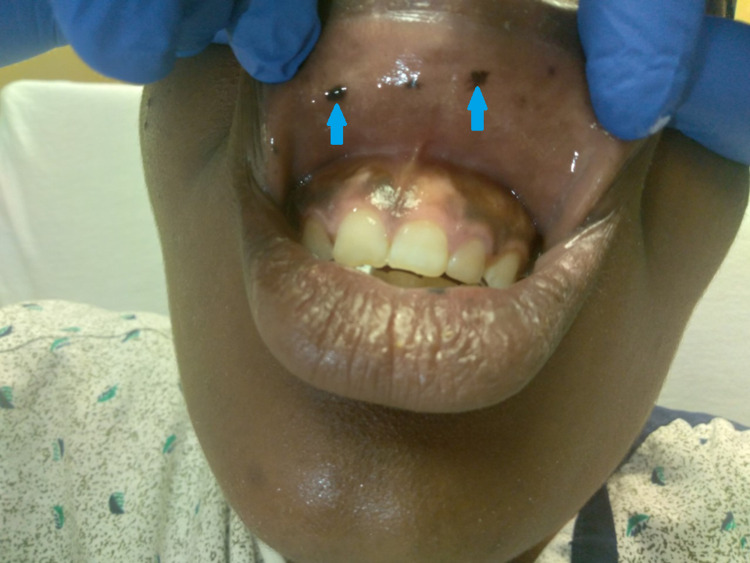
Mucocutaneous pigmented lesions on the upper oral mucosa

**Figure 3:**
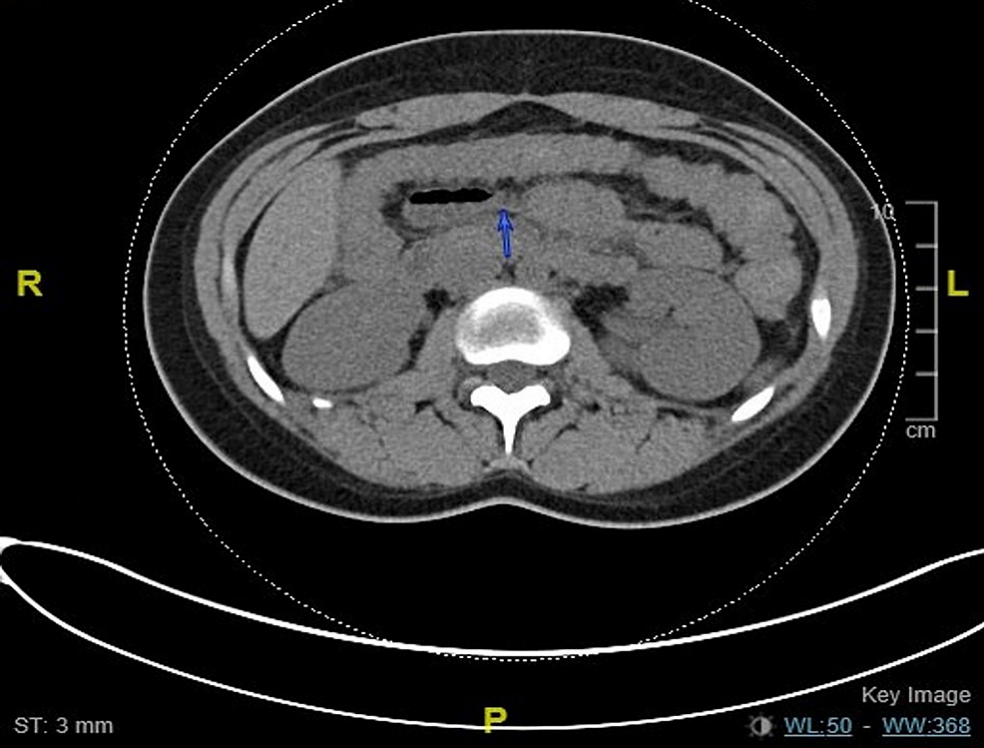
CT abdomen with the first transition point

A 20-year-old female presented to the hospital with substernal chest pain and intermittent abdominal pain. Physical examination was only remarkable for multiple pigmented macules on the buccal mucosa (Figure 1, 2). The patient was hemodynamically stable and saturating more than 95% on room air. Initial labs, including complete blood count (CBC), heartrate (BMP), and urinalysis, were remarkable for hemoglobin of 10.6 gm/dl. CT of the abdomen was concerning for small bowel intussusception with a lead point mass measuring 2 x 1.6 cm with no signs of obstruction (Figure 3). Surgery was consulted for evaluation of intussusception. The patient was subsequently taken for diagnostic laparoscopy and intraoperatively was noted to have four areas of intussusception involving the small bowel (Figure 5). The patient had a reported history of prior intussusception of the small bowel one year back, for which small bowel resection involving 7.5 cm of the small bowel, including an intraluminal mass through mini laparotomy, was performed. Pathology specimen at that point reported the mass to be Peutz-Jeghers polyp. Intraoperatively during this visit, reduction of the intussusception points was done without any bowel resection. The patient was subsequently discharged after postoperative day four with an unremarkable postoperative course. The patient later returned for an outpatient follow-up with an in-depth discussion about the patient's genetic condition and later presented to the hospital for elective diagnostic laparoscopy, exploratory laparotomy, and resection of the small bowel tumors, including resection of 10 cm of the small bowel. Pathology specimens were again sent which were consistent with Peutz-Jeghers polyps (Figure 6). The patient was discharged after an unremarkable postoperative course of four days.

**Figure 4:**
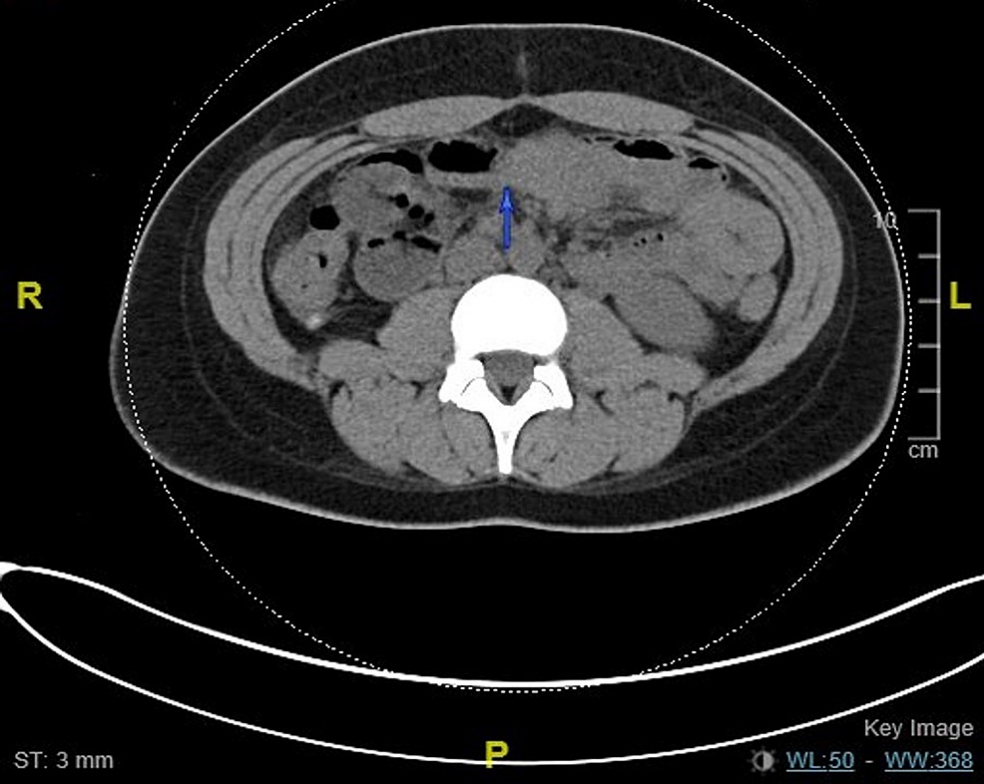
CT abdomen with the second transition point

**Figure 5:**
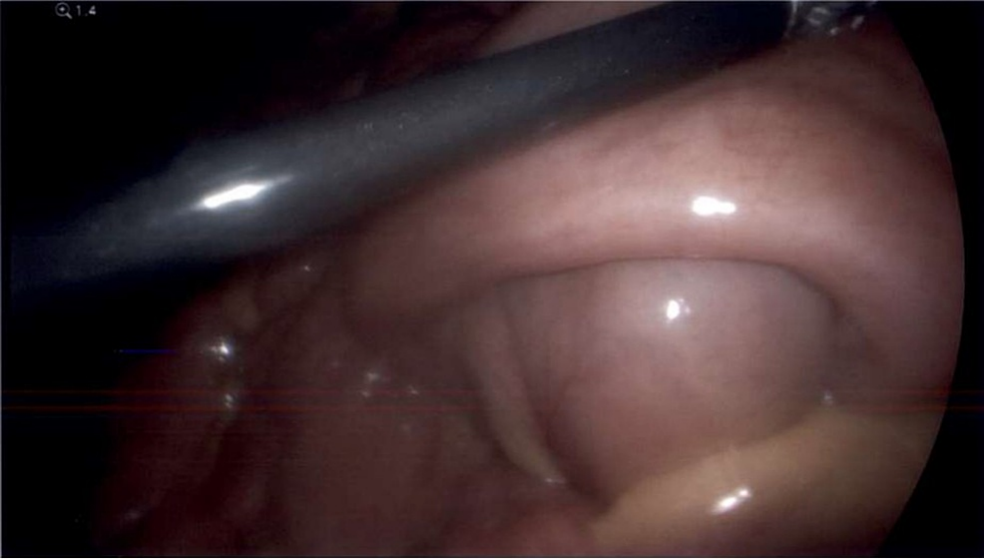
Intussusception of the small bowel

**Figure 6:**
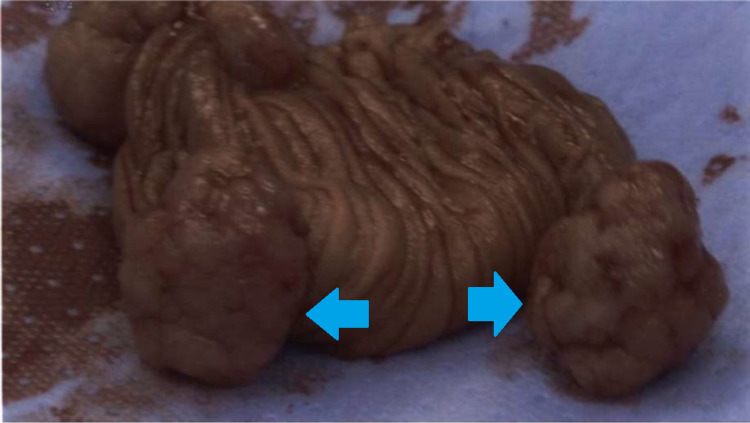
Hamartomatous Peutz-Jeghers polyps on resected small bowel specimen

## Discussion

PJS is an autosomal dominant disorder (1 per 200,000) with variable penetrance and is usually diagnosed at the age of 20-30 years. It is characterized by hamartomatous polyps in the small bowel (100%), stomach, and colon (25%), and the polyps may occur without other characteristic features of the syndrome, hence increasing the risk of intussusception and bleeding [8].

The patient presented above initially presented with one Peutz-Jeghers polyp; however, within a span of a year developed multiple polyps in the small intestine. This not only resulted in the patient having multiple points of intussusception but also increased her risk for bleeding and subsequent anemia. 

A clinical diagnosis of PJS can be made by the presence of any two of the following: two or more Peutz-Jeghers-type hamartomatous polyps of the gastrointestinal tract, mucocutaneous hyperpigmentation of the mouth, lips, nose, eyes, genitalia, or fingers, and a family history of PJS [9]. PJS is associated with an increased risk of multiple cancers, both gastrointestinal and extra-intestinal malignancies. In a systematic review of 20 observational studies studying about 1644 patients with PJS, the reported lifetime risk for any cancer varied between 37% and 93% [10]. The average age of developing a malignancy was 42 years.  The polyps may undergo a malignant change through the hamartoma-adenoma-carcinoma sequence, which warrants a close follow-up of the gastrointestinal tract and other solid tumors at risk. [11]. The most common sites for malignancy were noted to be colorectal, followed by breast, stomach, small bowel, and pancreas. It is uncertain whether the malignant lesions arise from hamartomas, associated adenomatous polyps, or normal mucosa [11]. 

Periodic surveillance and removal of larger polyps can decrease the likelihood of complications in Peutz-Jeghers syndrome. Hence, American College of Gastroenterology clinical guidelines recommend that patients should undergo an annual complete blood cell count and physical examination that includes evaluation of the breasts, abdomen, pelvis, and testes. Lifelong cancer surveillance is highly recommended [8]. Due to the increased risk for gastric, small bowel, and colorectal polyps and cancer in individuals with PJS, baseline endoscopic screening of the gastrointestinal tract should include upper gastrointestinal endoscopy, video capsule endoscopy (VCE), and colonoscopy, beginning at eight years old. Subsequent screening intervals should be based on the findings at baseline examination: if polyps are detected on baseline screening, upper endoscopy, VCE, and colonoscopy should be repeated every two to three years. If no polyps are detected on baseline screening, an upper endoscopy, VCE, and colonoscopy should be repeated at age 18 years, or sooner if symptoms arise, with repeat follow-ups at an interval of every two to three years [10]. 

Preventative screening is vital for Peutz-Jeghers syndrome patients in order to prevent further complications that stem from recurrent surgical interventions. 

## Conclusions

Peutz-Jeghers syndrome is an autosomal dominant genetic disorder characterized by mucocutaneous hyperpigmentation in the perioral and buccal mucosa and hamartomatous polyps in the gastrointestinal tract. As seen with the patient discussed in this case report, Peutz-Jeghers syndrome can lead to multiple complications, which include but are not limited to GI bleeding, bowel obstructions, rectal prolapse, and intussusception. Furthermore, the lifetime risk of malignancy significantly increases in patients with Peutz-Jeghers syndrome, most notably in the colorectal, breast, stomach, small bowel, and pancreatic regions. Inevitably, patients will almost always require surgical intervention to resect tumors or polyps associated with this condition. For this reason, screening for polyps in patients with a confirmed diagnosis of Peutz-Jeghers syndrome begins at the age of eight. In addition to annual physical examinations and annual complete blood cell counts, it is important to monitor the number and size of tumors in order to preemptively resect such tumors and prevent surgical complications. 
